# Strategies for Bacteriophage T5 Mutagenesis: Expanding the Toolbox for Phage Genome Engineering

**DOI:** 10.3389/fmicb.2021.667332

**Published:** 2021-04-26

**Authors:** Luis Ramirez-Chamorro, Pascale Boulanger, Ombeline Rossier

**Affiliations:** Université Paris-Saclay, CEA, CNRS, Institute for Integrative Biology of the Cell (I2BC), Gif-sur-Yvette, France

**Keywords:** bacteriophages, genome editing, CRISPR-Cas, retrons, dilution-amplification-screening

## Abstract

Phage genome editing is crucial to uncover the molecular mechanisms of virus infection and to engineer bacteriophages with enhanced antibacterial properties. Phage genetic engineering relies mostly on homologous recombination (HR) assisted by the targeted elimination of wild-type phages by CRISPR-Cas nucleases. These strategies are often less effective in virulent bacteriophages with large genomes. T5 is a virulent phage that infects *Escherichia coli*. We found that CRISPR-Cas9 system (type II-A) had ununiform efficacies against T5, which impairs a reliable use of CRISPR-Cas-assisted counterselection in the gene editing of T5. Here, we present alternative strategies for the construction of mutants in T5. Bacterial retroelements (retrons) proved to be efficient for T5 gene editing by introducing point mutations in the essential gene *A1*. We set up a protocol based on dilution-amplification-screening (DAS) of phage pools for mutant enrichment that was used to introduce a conditional mutation in another essential gene (*A2*), insert a new gene (*lacZ*α), and construct a translational fusion of a late phage gene with a fluorescent protein coding gene (*pb10-mCherry*). The method should be applicable to other virulent phages that are naturally resistant to CRISPR/Cas nucleases.

## Introduction

Bacteriophage genome engineering opens avenues to investigate the role of viral genes in infection and to generate bacteriophages with new properties. Until the late 1970s, genetic modifications were only possible through random mutagenesis (Drake and Baltz, 1976). Recombinant DNA technology later allowed the first phage gene editing to be carried out in T4 (Völker and Showe, 1980). Since then, continuous improvements of targeted mutagenesis methods were developed.

A first strategy, BRED (Bacteriophage Recombineering of Electroporated DNA), aimed to increase the recombination frequency between a template and a bacteriophage (Marinelli et al., 2008) but it relies heavily on the compatibility of the recombinases with the phage to be engineered and on a high electroporation efficiency, which is hard to achieve for phages with large genomes.

A second line of progress consisted in the improved recovery of phage mutants through the elimination of unmodified (wild-type) phages. In phage T7, selection of phage mutants was possible through the use of marker genes that are essential for phage infection in some host backgrounds (Grigonyte et al., 2020). Although the strategy looks promising, its application requires prior knowledge of host genes essential for infection, and the availability of suitable host mutants. This technique is therefore limited to well-studied phages in model organisms. An alternative to marker-based method is the use of CRISPR-Cas nucleases, enzymes that can be targeted to precise sequences thanks to their association with so-called guide RNAs. CRISPR-Cas nucleases were successfully used to eliminate the wild-type phages that have not undergone HR and thereby enrich the phage population in mutant viruses. Improved recovery of phage mutants was reported using type I-E, type II-A CRISPR or type III-A systems (Kiro et al., 2014; Martel and Moineau, 2014; Tao et al., 2017; Hatoum-Aslan, 2018; Hupfeld et al., 2018). Despite these improvements in the reverse genetic toolbox, some phages are recalcitrant to nucleases, either by inhibition (Acr proteins protecting the phages from CRISPR-Cas) (Marino et al., 2018) or by genome shielding (Malone et al., 2020; Mendoza et al., 2020).

Finally, a third line of progress consisted in performing the genome modifications *in vitro* followed by phage “rebooting,” where the entire phage genome is transformed into a recipient bacterium to yield viral particles (Ando et al., 2015; Kilcher et al., 2018). This bottom-up rebooting method requires a thorough optimization of transfection conditions and is very inefficient for phages with large genomes. Another avenue for rebooting could be the cell-free TXTL protein synthesis of phages *in vitro*: synthesis of T4 from the intact 170-kb genome T4 was recently reported, albeit with small yields (Rustad et al., 2018). Hence, no universal method seems to emerge for the editing of phages with large genomes.

Our laboratory studies bacteriophage T5, a virulent phage that infects *Escherichia coli* and has a linear 121-kb dsDNA genome. Most mutants in T5 were isolated in the past century after chemical mutagenesis and screening for amber mutations (Lanni et al., 1966; Hendrickson and McCorquodale, 1971). Since the 1980s, directed mutagenesis of T5 was also performed upon infection of host strains carrying a plasmid with the desired mutation. Usually ca. 500-bp homologous DNA is provided on each side of the mutation to favor HR. Screening of plaques was performed by PCR (Golomidova et al., 2016) or hybridization of radiolabeled oligonucleotides in stringent conditions (Vernhes et al., 2017; Klimuk et al., 2020), a rather cumbersome method. A recent report raised doubts about the possibility to use CRISPR-Cas for T5wt counterselection: T5 was reported to escape the CRISPR-Cas type I-E from *E. coli*. Accordingly, although most of the spacers incorporated into CRISPR arrays mapped to pre-early genes, only few of them conferred protection against T5 (Strotskaya et al., 2017). In this report, we tested the use of a Cas nuclease normally absent in *E. coli*, i.e., the type II-A nuclease Cas9, for restricting T5 infection. We also describe an alternative method based on a scheme of dilutions for improved recovery of mutants. Finally, we tested the use of retrons as another template for homologous recombination. These strategies likely constitute welcome additions to the toolbox for the phage genome engineering.

## Materials and Methods

### Bacteria, Phages, and Culture Media

The *E. coli* bacterial strains used in this work are described in Table 1. They were routinely grown at 37°C in LB broth (10 g tryptone, 5 g yeast extract and 5 g NaCl per liter) supplemented with 1.5% agar for solid media. When appropriate, antibiotics were used at the following concentrations: ampicillin, 200 μg/mL; chloramphenicol, 50 μg/mL kanamycin, 100 μg/mL. Prior to phage infections, cultures were supplemented with CaCl_2_ (1 mM) and MgCl_2_ (1 mM). T5 wild type (T5wt), T5 Δ*dmp*, T5 *lacZ*α, and T5 PNmC were propagated in *E. coli* F. T5 *amA1* SS84, T5 *amA1* T28, and T5 *amA2* S37 were amplified in *E. coli* CR63, which codes for an amber suppressor _*CUA*_tRNA^*Ser*^. Plaques were visualized on a double agar layer using molten LB agar (0.5%) supplemented with the indicator bacteria and the phages. For the blue/white screening of lysis plaques the molten top agar also contained X-Gal (0.6 mg/mL) and isopropyl β-D-1-thiogalactopyranoside (IPTG, 3 mM). For visualization of lysis plaques, plates were incubated at 37°C overnight and, when appropriate, a further incubation at 25°C for 24 h enhanced the blue color of the plaques.

**TABLE 1:** Plasmids, bacterial strains, and bacteriophages used in this study.

**Plasmids**	**References**
pCas9	Jiang et al., 2013
pUC19	Genbank: M77789.2
pBAD24	Guzman et al., 1995
pFF745	Farzadfard and Lu, 2014
psgRNAcos	Cui et al., 2018
pAC9	This study
pFFA1	This study
pUCamA1	This study
pUCDdmp	This study
pUCamA2	This study
pUC05-LacZa2	This study
pET-PNter-mCherry	Vernhes et al., 2017
pUC_PNmC	This study
***Escherichia coli* strains**	
DH5alpha	New England Biolabs
CR63	Appleyard et al., 1956
F	Lanni, 1958
MG1655	GenBank: U00096.3
XL-1 Blue	Bullock, 1987
**Bacteriophages**	
T5 wt	GenBank: NC_005859.1
T5 *amA1* strain SS84 (S84stop S85stop)	This study
T5 *amA1* strain T28 (T28stop)	This study
T5 *amA2* (S37stop)	This study
T5 Δ*dmp*	This study
T5 *lacZ*α	This study
T5 PNmC	This study

### Plasmid Constructions

Plasmid pAC9 was assembled from plasmids pCas9, pBAD24 and psgRNAcos (see Supplementary Table 1 and Supplementary Figure 1) by homologous recombination (Jacobus and Gross, 2015). Vectors were linearized by PCR amplification. PCR products were digested with *Dpn*I for 1 h at 37°C and purified. *E. coli* cells were transformed with 50 ng of each fragment and selected in LB agar with ampicillin or kanamycin.

For sgRNA cloning, sense and anti-sense oligonucleotides (Supplementary Table 2) were annealed using a temperature gradient from 98°C to 20°C at 0.1°C/s, and then cloned into pAC9 by the Golden-Gate method using the type IIs restriction enzyme *Bsa*I (Werner et al., 2012).

Plasmid pFFA1, used for retron-mediated mutagenesis, was obtained by PCR from the plasmid pFF745 with primers 3087 and 3127, and subsequent religation in a Golden-Gate reaction (Werner et al., 2012).

Plasmids used for HR were assembled with primers cited in Supplementary Table 1. In all cases, pUCGG was the recipient; this plasmid was obtained by PCR (with primers pUCF/pUCR) using pUC19 as the template, followed by religation. Inserts were amplified, purified and cloned in pUCGG by the Golden-Gate method. The QuikChange (QC) site-directed mutagenesis method was used to introduce specific mutations in the plasmids when needed (Deng and Nickoloff, 1992). pUCDdmp was constructed by cloning PCR products from T5 DNA (with primer pairs dmpF2/DdmpR and DdmpF/dmpR2). pUCA1am was constructed by cloning a PCR fragment amplified from T5 (using primers GGA1TF/GGA1TR) and the subsequent introduction of a stop codon (QCA11/QCA12) at the *A1* codon T28. For pUCA2am, the amplified insert (ggpUCA2F/ggpUCA2R) was cloned in pUGG and the *A2* codon S37 was then changed to stop codon (QCamA2F/QCamA2F). Plasmid pUC05-LacZa2 was constructed in two steps: the first insert in pUCGG bears a T5 genome fragment comprising gene *05* flanked by parts of genes *A1* and *A2*, amplified by PCR (05F/05R); the resulting plasmid, pUC05, was amplified (pUC05-F/pUC05-R) to clone *lacZ*α (LacZalpha-ATG/LacZalpha-Stop) from pUC19. The *Eco*RI site in *lacZ*α was erased (QC-LacZ-Eco1) and a Shine Dalgarno sequence was added (QCLacZSD1/QCLacZSD2) upstream to allow translation. For pUCPNmC construction, two inserts were fused, one from T5 (T5.151F/mCR) and the other one from pET-PNter-mCherry (mCF/T5.151R).

### CRISPR-Cas9 Interference Assay

Each host (*E. coli* strains F or CR63) bearing a pAC9 with or without the spacer against T5wt was grown at 37°C in LB supplemented with kanamycin, calcium, and magnesium until an OD_600_∼0.4–0.5 was reached. 100 μL of serial dilutions of the phage and 100 μL of the host were added to the top agar of a double agar overlay assay in presence of kanamycin as well as arabinose (0.4%) to induce the expression of Cas9. To measure infection interference, the host and phage were *E. coli* F(pAC9) and T5wt; for CRISPR-Cas9 assisted counterselection of T5wt following mutagenesis, *E. coli* CR63 carrying pAC9-derivatives was infected with the lysate from HR.

### Mutagenesis Using Plasmid Template for Homologous Recombination

The template plasmids used for phage mutagenesis are listed in Table 1. *E. coli* CR63 was transformed either with pUCA1am (to generate T5 *amA1*) or pUCA2am (for T5 *amA2*). *E. coli* XL-1 Blue was used along with pUC05-lacZa2 to generate T5 *lacZ*α, while *E. coli* F was transformed with pUCPNmC to generate T5 PNmC. Precultures of the cited hosts were started from a single colony inoculated in LB with ampicillin, shaken overnight at 37°C and diluted to OD_600_ ∼0.1 in LB broth supplemented with ampicillin, calcium and magnesium. Cultures were shaken at 37°C until they reached OD_600_ 0.4-0.5, and were then infected with T5 wt at MOI 10. After 10 min at 37°C, cells were washed twice through centrifugation at 5000 × *g* for 3 min and resuspension with LB broth supplemented with ampicillin, calcium and magnesium. Cells were harvested, resuspended at half of the initial volume, and incubated at 37°C for 90 min. Then 0.2% chloroform was added to the culture, and following 10 min at 37°C, the lysate was centrifuged at 5000 × *g* 3 min. The supernatant was filtered with 0.45-μm cellulose acetate filters (Sartorius) and stored at 4°C.

### Mutagenesis Using Retron

Overnight cultures of *E. coli* cells CR63(pFFA1) grown in LB supplemented with chloramphenicol were used to initiate a 20-mL culture at OD_600_ 0.1 in LB broth with chloramphenicol and 1mM each of IPTG, CaCl_2_ and MgSO_4_. The culture was shaken at 37°C and 220 rpm until the OD_600_ reached 0.4, and then T5wt was added at MOI 10. After 10 min at 37°C, cells were washed twice and resuspended in half the initial volume with LB supplemented with chloramphenicol, IPTG, calcium and magnesium. Following 90 min at 37°C, chloroform was added and cell lysates were harvested as described above for the mutagenesis using plasmid templates.

### Screening of Mutant Phages

For the PCR screening of phages obtained after mutagenesis, we performed a mismatched amplification mutation assay (MAMA-PCR) (Cha et al., 1992). The sequence of primers for the detection of T5 *amA1* (2772/A1Scr), T5 *amA2* (05R/4050.2), T5 *lacZ*α (LacZalpha-Stop/A2ATG), PNmC (mCF/104870) are shown in Supplementary Table 1. T5 Δ*dmp* mutants were screened with primers T5:14-38F and 03ATG (Supplementary Table 1), based on the PCR amplicon size. Thermocycler conditions were set to 98°C for 3 min, followed by 30 cycles at 55°C (30 s) and 72°C (1 min), and finally 5 min at 72°C. PCR reactions contained 10 μM of each desalted primer (Eurofins), DMSO 1.5%, 1 μL of filtered lysate, and Taq DNA Polymerase 0.05 U/μL with 1× Buffer with KCl (Thermo Fisher Scientific).

### Dilution-Amplification-Screening (DAS)

**FIGURE 1:**
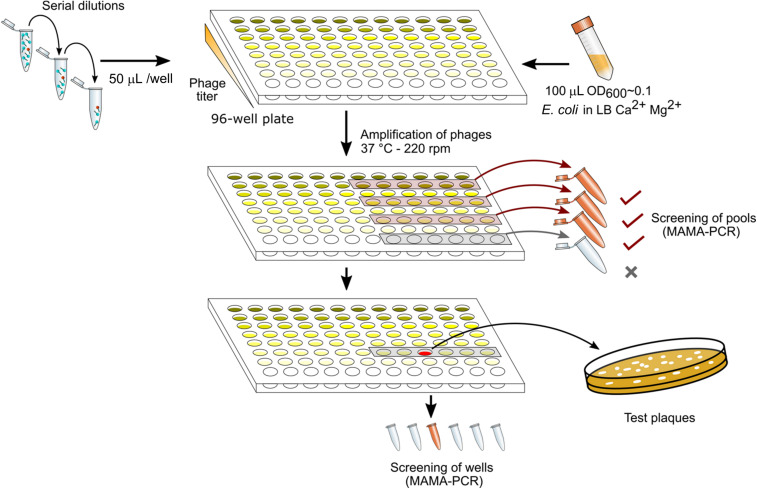
Phage mutant enrichment protocol by Dilution-Amplification-Screening (DAS).

Figure 1 summarizes the different steps in DAS. The lysate was first diluted by 10-fold serial dilutions to reach ca. 10–100 PFU/mL (usually up to a dilution factor of 10^8^ to 10^10^). Each dilution was performed in 10 mL of LB supplemented with CaCl_2_ (1mM) and MgSO_4_ (1mM). A total of 50 μL of each dilution were then distributed in twelve wells of a 96-well sterile flat bottom polystyrene plate (Corning), one row per dilution factor. To amplify the phages in each well, one-hundred-μL aliquots of a CR63 bacterial culture in exponential phase were added. The plate was incubated for 3 h at 37°C while shaking at 220 rpm. For the first screening by MAMA-PCR, we pooled 20 μL from six wells in the same row. Positive pools corresponding to the highest phage dilutions were selected, and the six individual wells were tested individually for the second MAMA-PCR screening. Finally, 50 μL taken from a PCR- positive well were plated on a double agar to test 10-20 lysis plaques individually.

### Fluorescence Microscopy

To observe the production of the late viral proteins by fluorescence microscopy, *E. coli* strain F was incubated at 37°C in LB supplemented with ampicillin, CaCl_2_ and MgCl_2_, until it reached an OD_600_ of 0.4. To synchronize the infections, the cells were chilled at 4°C for 15 min. Phage T5 PNmC was added to the culture at a MOI of 5 and incubated for 5 min at 4°C. The infected cells were washed by centrifugation at 5000 × *g* for 3 min and resuspension in LB. An aliquot of the culture was taken every 10 min and fixed with the same volume of PFA solution (Formaldehyde 5%, Glutaraldehyde 0.06%, diluted in PBS buffer) for 20 min. Fixed cells were washed in PBS buffer (NaCl 0.14 M, KCl 2.68 mM, Na_2_HPO_4_ 6.46 mM, KH_2_PO_4_ 1.15 mM) twice by centrifugation-resuspension. The cells were finally resuspended in PBS and stored at 4°C until microscopy imaging. Fixed cells were deposited on thin agarose pads (1% molecular-biology-grade agarose in PBS) molded on glass slide and observed under cover slips with a Zeiss Axio Observer Z1/7 microscope using the ZEN software, version 3.0. The objective was Plan Apochromat 63x/1.40 Oil Ph 3 M27. The exposure time was 200 ms for mCherry mPlum HXP (Ex: 587 nm, Em: 610 nm) and 20 ms for phase contrast.

## Results

### Restriction of T5 Infection by CRISPR-Cas9 Depends on the Targeted Locus

**FIGURE 2:**
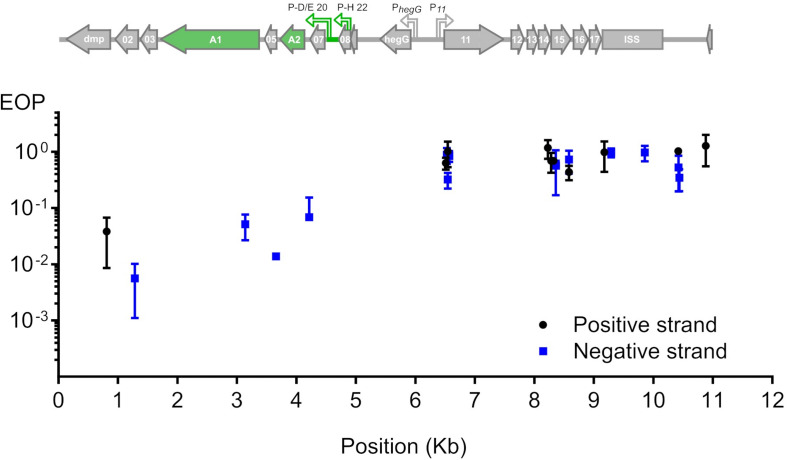
Restriction of phage T5 infection by sgRNA-guided Cas9. The restriction of T5 infection was measured as the efficiency of plating (EOP), calculated as the titer of T5 on sgRNA- and Cas9-containing hosts versus the titer on the same host lacking the sgRNA. Titers were determined by plaque assay. EOP using spacers matching the positive or negative strand were highlighted in black or blue, respectively. Each point represents the mean and s.e.m. from three replicas. Above the graph is the genetic organization of the first 11 kb from the 121-kb genome: green arrows highlight genes *A1* and *A2* which are predicted to be essential based on the phenotype of amber mutations genetically mapped in the 1960s.

To test whether phage T5 infection is restricted by CRISPR-Cas9, we transformed *E. coli* F with a plasmid coding for the nuclease Cas9 and customizable sgRNAs. The sgRNAs matched different locations across the first 10 kb of the T5 phage genome. By double-layer agar method, we compared the titer of a T5 stock on two host strains: *E. coli* F expressing both Cas9 and each sgRNA, versus the same host lacking the sgRNA. The ratio of PFUs between the two hosts constituted the efficiency of plating (EOP) and reflected the protective capacity of Cas9 against phages (Strotskaya et al., 2017; Tao et al., 2017) for each sgRNA. Restriction of T5 infection varied across the tested region regardless of the DNA strand that was targeted (Figure 2). Spacers with the highest interference mapped to the genes *dmp*, *02*, *A1*, *05*, and *07* and lowered the titer of T5wt between 10 and 100 times. The other spacers did not provide protection as the titer of T5wt decreased less than ten times. Therefore, Cas9 could be efficiently used to restrict T5wt infection when it was targeted to a region within the first 4.5 kb of the T5 genome.

### Bacteriophage T5 Genome Editing Through HR Plus CRISPR-Cas9

**FIGURE 3:**
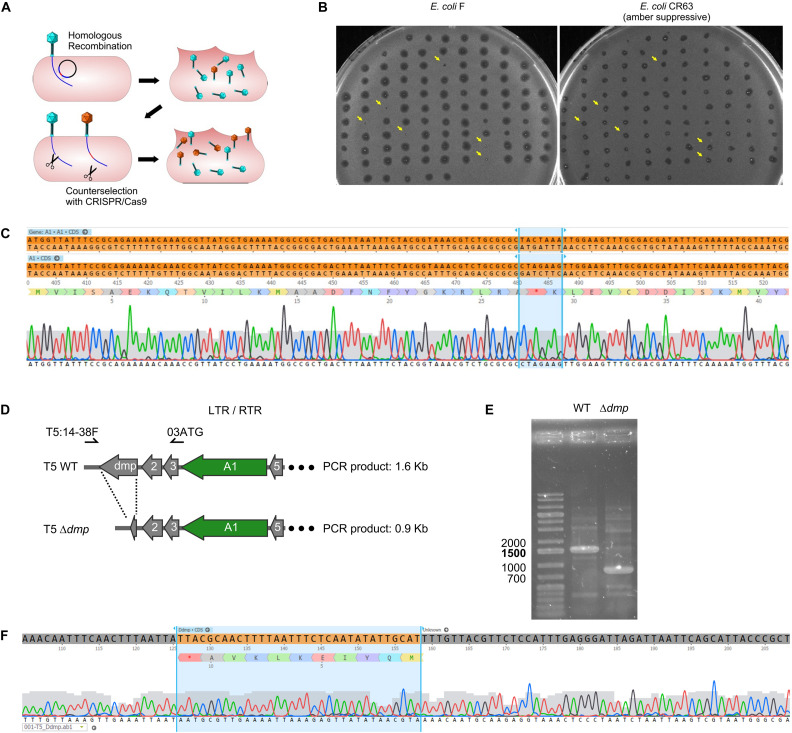
Construction of phage mutants by homologous recombination and mutant enrichment using sgRNA-guided Cas9. **(A)** Upon infection by wild-type phages of a host bearing a template plasmid, HR introduces the genetic modification in a subset of phages. In a second step, mutants among the progeny are enriched by counterselection onto a host producing the Cas9 nuclease targeted against the wild-type sequence. **(B)** Screening of the phages obtained after mutagenesis and counterselection. Lysis plaques were tested onto a non-permissive *E. coli* strain F and the amber suppressive strain CR63. Plaques with no lysis on *E. coli* F (yellow arrows) were considered positive and sequenced. **(C)** Sequencing results for one of the six amber mutants T5 *amA1* T28. Top lane corresponds to the T5 nucleotide sequence of wild type, the second lane shows the desired amber mutation within gene *A1* at position (3226.3332) of the T5 genome. Third lane corresponds to amino acid residues 16 to 50 translated from the sequence above. Fourth lane shows sequencing results. **(D)** Genetic map of pre-early genes *dmp*, *02*, *03*, *A1*, and *05* in wild-type T5 (above) and in T5 **Δ***dmp* (below). Primers used for PCR amplification T5:14-38F and 03ATG can anneal at both terminal repeats. **(E)** Fragments amplified by PCR using primers T5:14-38F and 03ATG and purified T5wt (WT), or purified T5 **Δ***dmp* mutant (**Δ***dmp*) phages. **(F)** Sequencing results for the T5 **Δ***dmp* depict the remaining ORF (start and last ten codons of *dmp*).

In order to introduce genetic modifications in T5, we focused on the region with effective spacers. In the late sixties, Lanni and coworkers described the isolation of T5 amber mutants that could only be propagated in an amber suppressive strain (Lanni et al., 1966; Lanni, 1968). Mutations were genetically mapped to *A1* or *A2* genes (Figure 2) but no sequencing data were obtained to confirm this finding. Therefore, we decided to introduce a stop codon early on in the open reading frame of the gene *A1*, resulting in the synthesis of truncated A1 (27 amino acids instead of 556). A 1-kb fragment of the T5 genome was amplified and cloned into a pUC19 plasmid. This fragment comprises 500-bp on each side of the *A1* start codon in order to provide ample homologous sequences on either side of the desired mutation, and the codon for T28 in the insert was replaced with a stop codon. Phage mutagenesis was performed by allowing infection of *E. coli* CR63 carrying the resulting plasmid. Counterselection of the T5wt within the crude lysate was carried out onto *E. coli* CR63(pAC9-A1), a strain that expressed Cas9 and a sgRNA directed against the wild-type *A1* gene at the same site where the amber mutation was introduced (Figure 3A). The crude lysate and CR63(pAC9-A1) were mixed prior to plating onto double agar plate. The resulting plaques were then picked and patched onto both *E. coli* strains F (non-permissive) and CR63 (amber-suppressive). We recovered six amber mutant plaques in 100 patched ones (Figure 3B). Sequencing confirmed the introduction of the intended mutation in the T5 *amA1* phages (Figure 3C).

The T5 genome has two long terminal direct repeats (ca. 10kb) that each include a copy of the 17 pre-early genes (Genome accession NC_005859.1). To test whether phages mutated in pre-early genes were modified on both terminal repeats, we performed a mutagenesis to delete the dispensable T5 pre-early gene *dmp* (Mozer et al., 1977). To provide a template for HR, we generated plasmid pUCDdmp, that carries a 702-bp deletion of *dmp* flanked by T5 sequences. For T5 mutagenesis, *E. coli* strain F (pUCDdmp) was first infected with T5 and then CRISPR-Cas9 counterselection of the crude lysate was done with *E. coli* F(pAC9_dmp) that produced a sgRNA directed against wild-type *dmp*. PCR screening of 100 plaques identified five plaques that bore the mutation (not shown). Using primers that anneal to both terminal repeats (Figure 3D), we could amplify a fragment corresponding to deleted version of *dmp* (Figures 3E,F). Therefore, the genome modifications were introduced in both terminal repeats. Taken together, our results demonstrate that it is possible to use sgRNA-guided Cas9 to facilitate mutant screening after HR in the genome of T5.

### Bacteriophage Genome Engineering With Retrons

**FIGURE 4:**
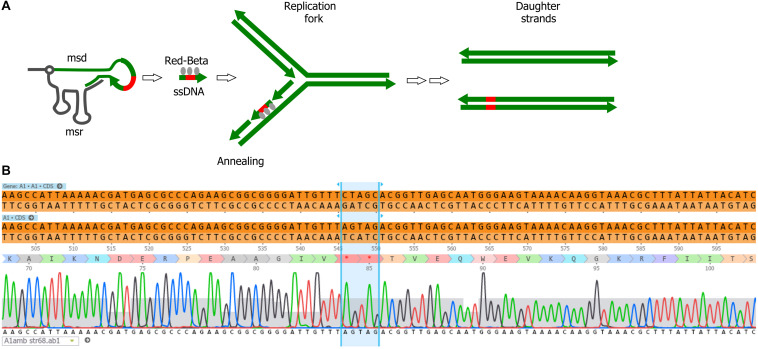
Retron-mediated mutagenesis. **(A)** Current model for retron-mediated mutagenesis. Retrons consist in covalently linked RNA (black line) and reverse-transcribed ssDNA (green). For mutagenesis purposes, retrons are modified to carry 75 nucleotides homologous to the target DNA (thick green line) with a mutation (red box). Thus, retrons provide the cell with a source of ssDNA, that together with the Red-Beta protein from phage lambda, anneals to the target DNA during replication. **(B)** Sequencing of the phage T5 *amA1* SS84 constructed by retron-mediated recombination: purified amber mutant phages T5 *amA1* SS84 obtained by retron-mediated mutagenesis were used as a template for PCR amplification. Amplicon sequences showed the presence of two stop codons instead of serine codons (S84 and S85) in the T5 *A1* gene.

We explored the use of alternative forms of DNA templates for phage genome engineering. Bacterial retroelements or retrons are chimeric RNA/DNA molecules composed of covalently linked ssRNA (msr) and ssDNA (msd). Both msr and msd are coded in the same cistron, altogether with a reverse transcriptase responsible for the partial reverse transcription and linking of the msr-msd molecules (Simon et al., 2019). These elements can provide ssDNA *in vivo* for gene modification, and were used for bacterial genome engineering in the past (Farzadfard and Lu, 2014; Simon et al., 2018; Figure 4A). We tested whether retrons are also effective for phage genome engineering. For the retron template, we modified plasmid pFF745, which codes for the msr, msd and the reverse transcriptase in a polycistron controlled by an IPTG inducible promoter (Farzadfard and Lu, 2014). Part of the msd sequence was replaced with a 75-bp segment of the viral *A1* gene centered on the serine codons for S84 and S85. These codons were substituted by stop codons, generating plasmid pFFA1. To introduce the mutation *A1* S84Stop/S85Stop into the phage genome, we grew *E. coli* CR63(pFFA1) in the presence of IPTG and infected the bacteria with the wild-type phage. For counterselection with Cas9, the crude lysate was plated onto *E. coli* CR63 bearing plasmid pAC9 and sgRNA matching the unmodified codons S84/S85 of T5 *A1*. Mutant screening was performed by picking 100 plaques and patching them onto plates seeded with *E. coli* strains F or CR63. We recovered 2 out of 100 plaques that could only lyse the amber-suppressing host strain. Such mutants were designated T5 *amA1* SS84. Sanger sequencing results showed the correct introduction of mutations in *A1* and no other alteration in the targeted locus (Figure 4B). Our results suggest that retrons could be effectively used for phage genome engineering.

### Dilution-Amplification-Screening to Isolate Mutants From Phage T5

**FIGURE 5:**
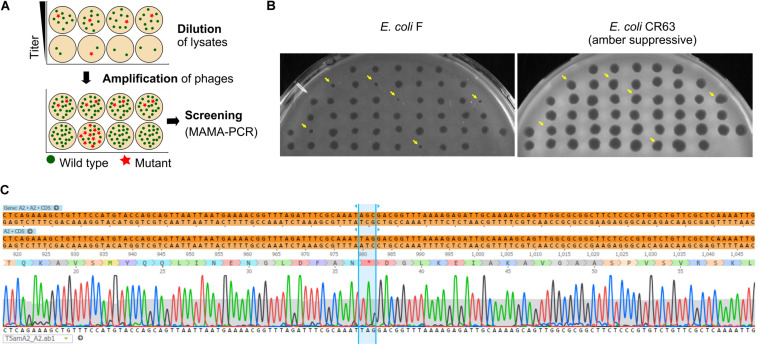
Screening of T5 mutants by Dilution-Amplification-Screening (DAS). **(A)** DAS principle: following mutagenesis, mutants are far less abundant than wild-type phages in the crude lysates. In the first step, dilutions of the lysates are performed to lower the phage titers and alter the proportion of mutants present in each pool (above, second row). In the second step, phages in the diluted pools are amplified by adding the host. While the mutants remain scant within amplified pools from initial high viral titers (below, first row), the modified phages become enriched in the progeny obtained with some of the highly diluted pools (below, second row). **(B)** Plaques obtained from a PCR-positive well were patched onto the non-permissive strain F and the amber-suppressive host CR63. Viruses that form plaques on the amber-suppressive strain only are highlighted with an arrow. **(C)** Sequencing result shows the correct introduction of point mutations in the T5 genome, resulting in a stop codon in the essential gene *A2*.

As we have seen above, some regions of the T5 genome are not amenable to the CRISPR-Cas9 counterselection, a feature that enriches the phage progeny in mutants relative to wild type after mutagenesis. We reasoned that a similar enrichment could be achieved with a simple dilution-amplification screening (DAS) (Figure 5A). As a proof of principle for this procedure, we sought to obtain a phage carrying an amber mutation in the essential gene *A2* at the codon S37. We allowed the recombination between the phage T5 and the plasmid bearing a mutated copy of the gene *A2* during infection. Following filtration to eliminate uninfected bacteria, the crude lysate was serially diluted from ca. 10^10^ PFU/mL down to 10^2^ PFU/mL. We distributed each dilution into several wells containing the amber-suppressive host and incubated for 3 h to amplify the phages (Figures 1, 5A). We screened the wells by mismatched amplification mutation assay (MAMA)-PCR to detect the mutation and the positive wells were plated by a double agar layer method to obtain individual plaques. The plaques were picked and patched successively onto two plates with a non-permissive and a permissive host, respectively. The plaques that lysed only the permissive host were considered positive (Figure 5B). We recovered 6 positives mutants from 50 tested, from which one was confirmed by Sanger sequencing (Figure 5C). Since our estimation of T5 recombination frequency is around 0.25% (see below), our recovery rate of 12% indicates that we could enrich the mutant in some pools enough to simplify the recovery of T5 *amA2* S37 mutants. Hence, DAS is an efficient method for mutant isolation.

### Blue T5: Insertion of lacZα Gene Into the T5 Genome

**FIGURE 6:**
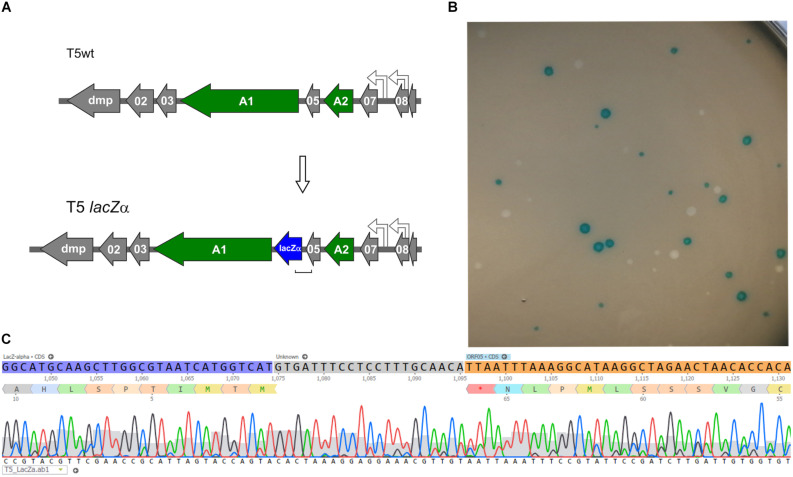
Construction of a Blue T5 (T5 *lacZ***α**) phage. **(A)** Genetic maps showing that the gene *lacZ***α** (blue arrow) was introduced between genes *A1* and *05* in the genome of T5. **(B)** Plaques of T5 *lacZ***α** and T5wt obtained after they were plated together with *E. coli* XL-1 Blue on a soft agar overlay supplemented with X-Gal and IPTG: only the mutant produced blue plaques. **(C)** Sequencing results of one of the blue plaques, showing the correct insertion of *lacZ***α** downstream of gene *05.*

Following the successful introduction of point mutations in the T5 genome, we further tested whether insertion of exogenous genes was possible. To generate a T5 phage that produces blue lysis plaques, a 270-bp ORF encoding the fragment LacZα of the *E. coli* β-galactosidase was introduced by HR between the genes *05* and *A1* and DAS was used to recover mutants (Figure 6). The T5 *lacZ*α (Blue T5) plaques were blue when plated in a soft agar overlay with the strain *E. coli* XL-1 Blue in the presence of X-Gal and IPTG (Figure 6B). When we skipped the DAS procedure and relied only on the blue/white plaque screening, we were able to measure the frequency of recombination in T5 by HR. We counted the number of blue lysis plaques obtained from the filtered crude lysate and found that 0.27 ± 0.01% (*n* = 6) of the phages were recombinants, i.e., had integrated the *lacZ*α gene. In T4, the frequency of recombinant phages recovered following infection of cells carrying the template DNA on a plasmid was reported to be between 0.1 and 0.5% (Völker and Showe, 1980), while in T3, it is comprised between 0.1 and 1% (Yehl et al., 2019). Therefore, the frequency of HR in T5 is consistent with that of other phages from the T series.

### Phages Producing a Chimeric Fluorescent Late Protein

**FIGURE 7:**
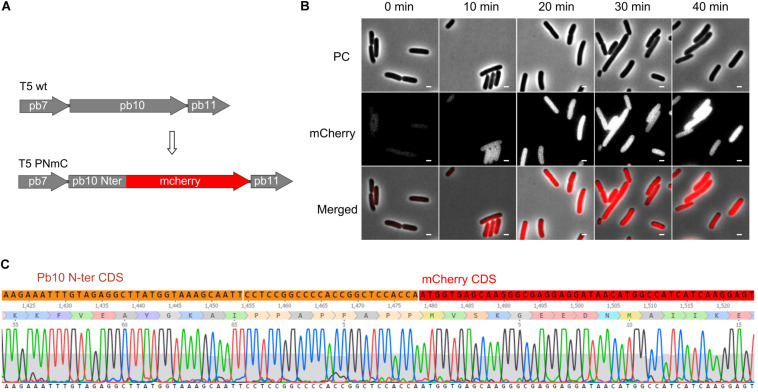
Gene fusion encoding a chimeric protein between the first half of the late protein Pb10 and a fluorescent protein. **(A)** Map of a segment of the capsid gene locus in the genome of T5 PNmC mutant: the 3’ end of the gene encoding Pb10 was replaced by a sequence encoding mCherry. The chimeric gene *pb10*(Nter)-*mcherry* (**Δ** [103638.103928]:*mcherry*) replaces the wild-type pb10 gene. **(B)** Phase contrast (PC), and fluorescence (mCherry) microscopy of *E. coli* F cells infected by T5 PNmC. The time after infection is indicated above. White bars, 2 μm. **(C)** Sequencing results of the Pb10-encoding gene in T5 PNmC showing the region where the Pb10 Nter coding sequence (in orange) merges with the *mcherry* ORF (in red).

To test whether our mutagenesis protocol was applicable to another region of the T5 genome, we generated a gene fusion between part of gene *151* and a reporter gene encoding a fluorescent protein. The product of T5 gene *151*, the late protein pb10, is a two-domain protein that decorates the surface of the T5 capsid. Although dispensable for capsid assembly, it contributes to its stability under high temperature (Vernhes et al., 2017). The N-ter domain of pb10 that binds to specific sites on the capsid was fused to mCherry (Figures 7A,C). Mutant phages were recovered and could be used to monitor the timing of late protein production during infection by fluorescence microscopy. There was a rapid rise of red fluorescence inside the cells after around 20 min of infection (Figure 7B), consistent with the expected late synthesis of the PNmC fusion.

## Discussion

In this work, we have edited the genome of bacteriophage T5 and could generate mutants carrying point mutations, a deletion or insertions. Template DNA for mutagenesis was provided on a plasmid with ca. 500-bp homologous DNA on either side to facilitate homologous recombination, a classical method used for the genome engineering of many phages (Pires et al., 2016). Here, we also chose to test an alternative template DNA, i.e., bacterial retroelements called retrons. Retrons code for mixed, covalently linked molecules constituted by single-stranded RNA and DNA, msd-msr, which can provide ssDNA molecules *in vivo*. They were successfully used for gene editing of *E. coli* cells expressing the lambda recombinase Red-Beta (Simon et al., 2019). Although the mechanism of mutagenesis by msd-msr molecules remains unknown, it has been suggested that the ssDNA produced from msd-msr cleavage replaces Okasaki fragments during replication leading to the introduction of modifications in the genome (Farzadfard and Lu, 2014). Retrons were used in the past to modify the bacterial genome upon exposure of the cells to certain stimuli (Farzadfard and Lu, 2014) or to continuously target specific sequences in the bacterial genome to drive evolution (Simon et al., 2018). To our knowledge, our report is the first example of phage genome editing using retrons. Since the completion of this work, an article described that some retrons, including the Ec86 retron used in this study, provide protection against T5 infection, albeit in a different *E. coli* strain background (Millman et al., 2020). Because the mechanism of host defense provided by Ec86 retron was not studied further, it is not clear whether the replacement of 75 nucleotides in *msd* by a heterologous sequence is enough to inactivate the retron-mediated protection. Therefore, prior to undertaking retron-mediated mutagenesis, regardless whether the hosts are Gram-negative or Gram-positive bacteria, it might be useful to test the effect of retron expression on the outcome of phage infection. In our study, the small size of the homologous domain (75-bp) is incompatible with insertions of large sequences in the genome but was sufficient to successfully introduce point mutations in T5 (here two stop codons). Nevertheless, mutagenesis with retrons offers several advantages. First, directed mutagenesis of cloned fragments is not necessary since the retron’s small size allows the cloning of annealed oligonucleotides carrying the desired mutations. Second, unlike the classical templates for HR, there is no need to provide long sequences flanking the mutations which might encode toxic products for the host cell. Further work will test whether retrons can also be used to construct gene deletions in phages.

Reported phage homologous recombination frequencies vary widely from 10^–10^ to 10^–2^ depending on the phages and the genes that are targeted (Pires et al., 2016). These rates warrant efficient screening strategies of the phage progeny after mutagenesis. One common approach is the targeted elimination of wild-type phage using interference by CRISPR-Cas. Mutant phages are thus enriched within the phage population (Kiro et al., 2014; Martel and Moineau, 2014; Tao et al., 2017; Hupfeld et al., 2018). Using Cas9 and sgRNAs targeting genes within the first 4.5 kb of T5 DNA, we were successful in isolating conditional mutants in the essential gene *A1* and a mutant deleted in the dispensable gene *dmp*. Interestingly, PCR amplification showed that the latter mutant was modified on both direct terminal repeats. Although the mechanisms for T5 replication are poorly understood, duplication of a terminal repeat during the packaging of concatemeric DNA has been proposed (Casjens and Gilcrease, 2009). We speculate that, in the case of a duplicated gene located outside of the terminal repeats, the mutagenesis outcome would not have necessarily led to the modifications of both genes.

Despite some success with sgRNA-guided Cas9, we found that interference was not uniform when we targeted different locations across the pre-early gene locus, something similar to what was been seen in T4 (Tao et al., 2017). While T4 DNA is partially protected through the modification of cytosines with 5-hydroxymethylation plus glycosylation, no modification of T5 DNA has been reported so far. This resistance to CRISPR-Cas interference was not limited to Cas9 nuclease: indeed a recent study of the impact of CRISPR-Cas type I-E against T5 showed that the few efficient spacers matched pre-early gene sequences but not the rest of the genome (Strotskaya et al., 2017). These observations suggest that there might be a generalized way, other than base modification, in which T5 manages to escape CRISPR-Cas nucleases. A similar observation in jumbo phages led to the discovery of a viral proteinaceous shell that protects phage DNA from nucleases (Malone et al., 2020; Mendoza et al., 2020). However, whether phage T5 uses a similar mechanism is unknown.

For phages resistant to CRISPR-Cas interference, we have established an alternative enrichment method by Dilution, Amplification, and Screening (DAS). The strategy relies on the dilution of the viral progeny into parallel pools, followed by their amplification in the host. Screening of the most dilute pools occurs using a discriminative PCR. This method was successful in isolating conditional as well as insertion mutants of bacteriophage T5. In this work we generated a bacteriophage mutant useful to detect late viral gene expression in fluorescence microscopy of *E. coli* infected by T5. We also used DAS to generate a phage carrying a 270-bp *lacZ*α gene among the early genes of T5. This phage was easily trackable when infecting *E. coli* cells designed for alpha complementation of beta-galactosidase activity, since its blue plaques are easily distinguished from colorless plaques. Using this phenotype, we were able to determine the recombination frequency of T5, which is similar to that of T3 (Yehl et al., 2019) and T4 (Völker and Showe, 1980). Such an easily trackable phage could prove useful in competition assays with other phages in the future. Finally, we believe that DAS can document that some genes targeted for mutagenesis are essential for infection, as the phages carrying the mutation after HR will not propagate during the amplification step and therefore will only be abundant in the least diluted pools. For this type of analysis, the PCR reactions should only allow the detection of the mutation within the phage genome and not the one provided on the plasmid DNA used as template.

Taken together, our work has shown that retrons and DAS were successful tools in the mutagenesis of T5, a phage with a large genome mostly recalcitrant to CRISPR-Cas nucleases. These tools are likely welcome additions to the biotechnological toolkit for phage genome editing.

## Data Availability Statement

The raw data supporting the conclusions of this article will be made available by the authors, without undue reservation.

## Author Contributions

LR-C, PB, and OR contributed to conception and design of the study, and analyzed the data. LR-C performed the experiments, developed the methods, and acquired the data. LR-C and OR wrote the first draft of the manuscript. All authors contributed to manuscript revision, read, and approved the submitted version.

## Conflict of Interest

The authors declare that the research was conducted in the absence of any commercial or financial relationships that could be construed as a potential conflict of interest.
