# Supplementary material for: Strategies for Bacteriophage T5 Mutagenesis: Expanding the Toolbox for Phage Genome Engineering
Source: Front Microbiol. 2021 Apr 26;12:667332. doi: 10.3389/fmicb.2021.667332 (PMC8108384; doi:10.3389/fmicb.2021.667332)
Supplement: Supplementary file 1 [file Data_Sheet_1.docx]

Supplementary materials

Table S1. Oligonucleotides used for cloning or mutagenesis

| Construction | Primer | Sequence |
| --- | --- | --- |
| pAC9 | 2225 | tttttgggctaacaggaggaattaaccATGGATAAGAAATACTCAATAGGCTTAGATATCGGCAC |
|  | 6331 | gaaaatcttctctcatccgccaaaacaGCCTCAGTCACCTCCTAGCTGACTCAAATCAATG |
|  | 2225c | CATGGTTAATTCCTCCTGTTAGCCCAAAAA |
|  | 6331c | GGCTGTTTTGGCGGATGAGAGAAGATTTT |
|  | Cas9F | gcctttttacggttcctggcACGAAGCAGGGATTCTGCAA |
|  | Cas9R | cagctcactcaaaggcggtaAGGGTTATTGTCTCATGAGCGG |
|  | psgF | TACCGCCTTTGAGTGAGCTG |
|  | psgR | GCCAGGAACCGTAAAAAGGC |
|  | QCpBADF | GTGTGGGGTCACCCCATGCGAG |
|  | QCpBADR | CTCGCATGGGGTGACCCCACAC |
|  | ACYCoriF | GTAAAAAGGCCGCGTTGCTGGCGTTGCGCTAGCGGAGTGTATACTGGC |
|  | ACYCoriR | CCGTAGAAAAGATCAAAGGATCTTCACAACTTATATCGTATGGGGCTGACTTCAGG |
|  | BC9GF | GAAGATCCTTTGATCTTTTCTACGGGGTCTGA |
|  | BC9GR | AACGCCAGCAACGCGGC |
| pUC19 *^b^* | pUCF | [Phos]-aaaaggtctcattttCTGGCCGTCGTTTTACAACG |
|  | pUCR | aaaaggtctcaggggTCATGGTCATAGCTGTTTCCTGT |
| pUCDdmp *^a^* | dmpF2 | aaaaggtctcaaaaaGCGGGAGTTTTCGGAATGACT |
|  | dmpR2 | aaaaggtctcaccccCGAGGCCGGTCACGTTATTG |
|  | DdmpF | aaaaggtctcacattTTGTTACGTTCTCCATTTGAGG |
|  | DdmpR | aaaaggtctcaaatgCAATATATTGAGAAATTAAAAGTTGCGTAATAATTAAAG |
|  | T5:14-38F | CGGCGAAAATACTTAGTCTGCTAAC |
|  | 03ATG | aaaaggtctcatcacATGGCTATTAAAATTAATCTTCCCAGCAT |
| T5 *amA1* | 3087 | aaaaggtctcaGTAGACGGttgAGCAATGGGAAGTAAAACAAGGTAAACGGAATTCAGGAAAACAGACAGTAACTCA |
| SS84 *^a^* | 3127 | aaaaggtctcaCTACTAAACAATCCCCGCCGCTTCTGGGCGCTCATCGTTTGAATTCAGGAAACCCGTTTTTTCTG |
|  | 3106 | CCCATTGCTCAACCGTCTACT |
|  | gRNA-A1S | tagtGGCGGGGATTGTTTCTAGCA |
|  | gRNA-A1A | aaacTGCTAGAAACAATCCCCGCC |
| T5 *amA1* | GGA1TF | aaaaggtctcaccccAGACCTTCAAGATTCAGCGCG |
| T28 *^a^* | GGA1TR | aaaaggtctcaaaaaCACTGGAAAGTGCAAAATTGGAAAC |
|  | QCA11 | ATATCGTCGCAAACTTCCAActtctAGGCGCGCAGACGTTTACCGTA |
|  | QCA12 | TACGGTAAACGTCTGCGCGCCTagaagTTGGAAGTTTGCGACGATAT |
|  | 2772 | AGACCTTCAAGATTCAGCGCG |
|  | A1Scr | CGGTAAACGTCTGCGCGCCTagaag |
|  | gRNAA1TS | tagtACGTCTGCGCGCTACTAAAT |
|  | gRNAA1TA | aaacATTTAGTAGCGCGCAGACGT |
| T5 *amA2* | gg pUC A2 F | aaaaggtctcaccccCGGCGGCAATAAAACAAATC |
| S37 *^a^* | gg pUC A2 R | aaaaggtctcaaaaaCAGGTAATGCGGAACAATCC |
|  | QCamA2F | ATCTCTTTTAAACCGTCctaATTTGCGAAATCTAAACC |
|  | QCamA2R | GGTTTAGATTTCGCAAATtagGACGGTTTAAAAGAGAT |
|  | 4050.2 | GAAAACGGTTTAGATTTCGCAAAgtag |
| T5 *lacZ*α *^a^* | 05F | aaaaggtctcaaaaaACCGCAAAATTCGCTTGGAA |
|  | 05R | aaaaggtctcaccccCTAACCAGCAAATCAGCGCC |
|  | pUC05-F | aaaaggtctcaTAGGCGTTATAATTTGCAACATTAATTTAAAGGCATAAGG |
|  | pUC05-R | aaaaggtctcaATTCAAGGCGTTATATTTGGCAATATTGCCAATAAC |
|  | LacZalpha-ATG | aaaaggtctcacctaATGACCATGATTACGCCAAGCTTGC |
|  | LacZalpha-Stop | aaaaggtctcagaatCTATGCGGCATCAGAGCAGATTGTAC |
|  | QC-LacZ-Eco1 | GGATCCCCGGGTACCGAGCTCAAACTCACTGGCCGTCGTTTTACAA |
|  | QC-LacZ-Eco2 | TTGTAAAACGACGGCCAGTGAGTTTGAGCTCGGTACCCGGGGATCC |
|  | QCLacZSD1 | GCAAGCTTGGCGTAATCATGGTCATgtGattTccTccTTTGCAACATTAATTTAAAGGCATA |
|  | QCLacZSD2 | TATGCCTTTAAATTAATGTTGCAAAggAggAaatCacATGACCATGATTACGCCAAGCTTGC |
|  | A2ATG | aaaaggtctcatcacATGACTAACGCTAAAACCGCAAAATTC |
| T5 PNmC *^a^* | T5.151F | aaaaggtctcaccccTGACCATTCATGCTCTTAGCGA |
|  | T5.151R | aaaaggtctcaaaaaTGCTTGATGGTGGTAATAATGCG |
|  | mCF | aaaaggtctcaattaAGACTTGTACAGCTCGTCCAT |
|  | mCR | aaaaggtctcataatGGAGGCTAATAAATGACACAAGC |
|  | 104870 | GGATAACAGTTACGTGTGTGGC |

Upper-case letters match with the *^a^* T5 genome or *^b^* plasmid pUC19.

Table S2. Oligonucleotides to generate the sgRNA for the CRISPR-Cas9 Infection Interference Assay

| Name *^a^* | Plasmid | Sense (S) | Anti-Sense (A) | Strand | Locus | EOP | SD |
| --- | --- | --- | --- | --- | --- | --- | --- |
| gdmp | pAC_dmp | tagtGGGAAATATGCGGGAAATTA | aaacGTAATTTCCCGCATATTTCC | Pos | 829 | 3.81x10^-2^ | 2.95x10^-2^ |
| gRNA-02 | pAC_02 | tagtCGCTCAGTCTTCTATTGTTC | aaacGAACAATAGAAGACTGAGCG | Neg | 1283 | 5.61x10^-3^ | 4.50x10^-3^ |
| gRNAA1T | pAC_A1T | tagtACGTCTGCGCGCTACTAAAT | aaacATTTAGTAGCGCGCAGACGT | Neg | 3289 | 4.22x10^-1^ | 3.19x10^-1^ |
| gRNA05 | pAC_05 | aaacAGAATGAGGGATTCAGTAGG | tagtCCTACTGAATCCCTCATTCT | Neg | 3658 | 1.38x10^-2^ | 1.93x10^-3^ |
| g07inv | pAC_07inv | tagtTATATCCCCTAGTAGTTCGG | aaacCCGAACTACTAGGGGATATA | Neg | 4217 | 6.29x10^-2^ | 9.03x10^-2^ |
| g10 | pAC_10 | tagtGCAACCTGCTGGATGGTAGC | aaacGCTACCATCCAGCAGGTTGC | Pos | 6563 | 1.02x10^+0^ | 4.91x10^-1^ |
| g10.4 | pAC_10.4 | tagtAGATAACGCAACCGTCTTAA | aaacTTAAGACGGTTGCGTTATCT | Pos | 6512 | 6.31x10^-1^ | 1.50x10^-1^ |
| g10.5 | pAC_10.5 | tagtTTTAAGACGGTTGCGTTATC | aaacGATAACGCAACCGTCTTAAA | Neg | 6532 | 8.87x10^-1^ | 2.79x10^-1^ |
| g10.6 | pAC_10.6 | tagtGCGCAAGGCGCCCTTTAAGA | aaacTCTTAAAGGGCGCCTTGCGC | Neg | 6545 | 3.21x10^-1^ | 9.93x10^-2^ |
| g14.5 | pAC_14.5 | tagtTTTCGTGGAGTGTTATAAAA | aaacTTTTATAACACTCCACGAAA | Pos | 8281 | 6.89x10^-1^ | 2.64x10^-1^ |
| g14.7 | pAC_14.7 | tagtCTTTGTTGCTATTTGCCGAC | aaacGTCGGCAAATAGCAACAAAG | Pos | 8323 | 6.90x10^-1^ | 7.47x10^-2^ |
| g14.6 | pAC_14.6 | tagtAAGTAGTTTGCTAATAATCC | aaacGGATTATTAGCAAACTACTT | Neg | 8364 | 5.66x10^-1^ | 5.87x10^-2^ |
| g14.3 | pAC_14.3 | tagtCGCATATATTCAGTCAACTG | aaacCAGTTGACTGAATATATGCG | Pos | 8584 | 4.37x10^-1^ | 1.24x10^-1^ |
| g14.4 | pAC_14.4 | tagtGCCTTTCATTATCAGGCGCT | aaacAGCGCCTGATAATGAAAGGC | Neg | 8584 | 7.28x10^-1^ | 3.19x10^-1^ |
| gIS3 | pAC_IS3 | tagtAACGGGGAGCTGATCCCCGT | aaacACGGGGATCAGCTCCCCGTT | Neg | 9293 | 9.73x10^-1^ | 1.73x10^-1^ |
| gIS7 | pAC_IS7 | tagtGCACACTATAAAAATTTTTC | aaacGAAAAATTTTTATAGTGTGC | Neg | 9859 | 9.74x10^-1^ | 2.98x10^-1^ |
| gIS6 | pAC_IS6 | tagtTGTATTAAACCGCCTATTGC | aaacGCAATAGGCGGTTTAATACA | Pos | 10421 | 1.03x10^+0^ | 1.28x10^-1^ |
| gIS5 | pAC_IS5 | tagtCAGGGCGGTTATAGGGAGGG | aaacCCCTCCCTATAACCGCCCTG | Neg | 10422 | 5.25x10^-1^ | 3.29x10^-1^ |
| gIS4 | pAC_IS4 | tagtATAGGCGGTTTAATACAGGG | aaacCCCTGTATTAAACCGCCTAT | Neg | 10437 | 3.45x10^-1^ | 1.47x10^-1^ |

*^a^* Add the character S or A at the end of the primer to get the primer's name.


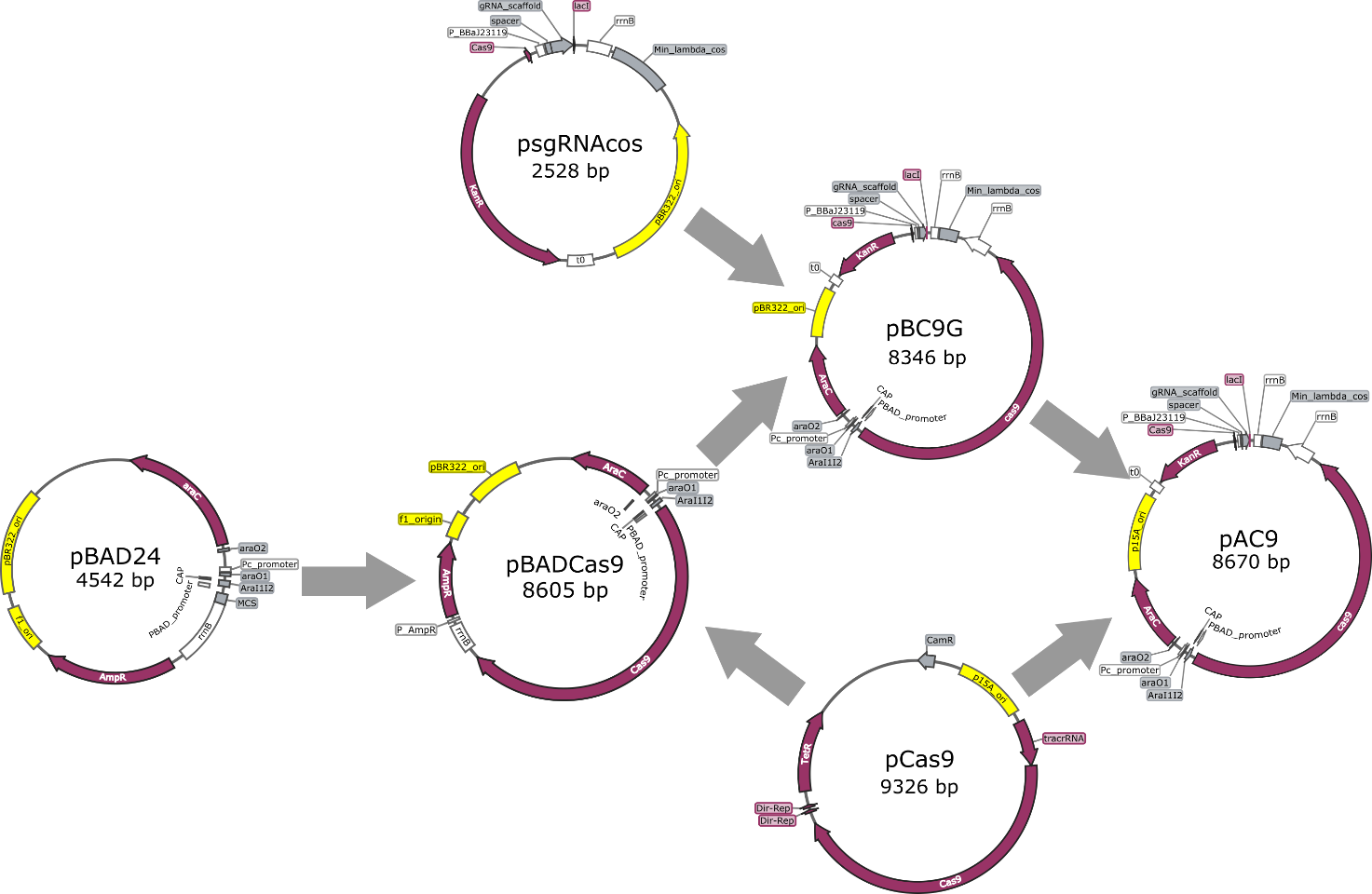


Figure S1. Construction of plasmid pAC9. The plasmid pBAD24 was amplified by PCR with primers 2225c/6331c to clone the gene *cas9* taken from the plasmid pCas9 with primers 2225/6331. pBADCas9, amplified with Cas9F/R, was fused with the sgRNA coding fragment of psgRNAcos. psgRNAcos was amplified with primers psgF/R. The origin of replication pBR322, from pBC9G, was replaced by the origin of p15A from pCas9 to obtain a mid-copy number plasmid: p15A was amplified with primers ACYCoriF/R, while the plasmid pBC9G was amplified by PCR with primers BC9GF/R.
